# SNP and Structural Study of the Notch Superfamily Provides Insights and Novel Pharmacological Targets against the CADASIL Syndrome and Neurodegenerative Diseases

**DOI:** 10.3390/genes15050529

**Published:** 2024-04-23

**Authors:** Louis Papageorgiou, Lefteria Papa, Eleni Papakonstantinou, Antonia Mataragka, Konstantina Dragoumani, Dimitrios Chaniotis, Apostolos Beloukas, Costas Iliopoulos, Erik Bongcam-Rudloff, George P. Chrousos, Sofia Kossida, Elias Eliopoulos, Dimitrios Vlachakis

**Affiliations:** 1 Laboratory of Genetics, Department of Biotechnology, School of Applied Biology and Biotechnology, Agricultural University of Athens, 75 Iera Odos, 11855 Athens, Greece; louis.papageorgiou@aua.gr (L.P.); lefteria.papa@aua.gr (L.P.); eleni@aua.gr (E.P.); antonia.mataragka@gmail.com (A.M.); kwstantina@gmail.com (K.D.); eliop@aua.gr (E.E.); 2 Department of Biomedical Sciences, School of Health and Care Sciences, University of West Attica, Agioy Spyridonos, 12243 Egaleo, Greece; dchaniotis@uniwa.gr (D.C.); abeloukas@uniwa.gr (A.B.); 3 University Research Institute of Maternal and Child Health & Precision Medicine, National and Kapodistrian University of Athens, “Aghia Sophia” Children’s Hospital, 11527 Athens, Greece; chrousos@gmail.com; 4 School of Informatics, Faculty of Natural & Mathematical Sciences, King’s College London, Bush House, Strand, London WC2R 2LS, UK; csi@kcl.ac.uk; 5 Department of Animal Biosciences, Swedish University of Agricultural Sciences, 756 51 Uppsala, Sweden; erik.bongcam@slu.se; 6 IMGT^®^, The International ImMunoGenetics Information System^®^, Laboratoire d’ImmunoGénétique Moléculaire LIGM, Institut de Génétique Humaine, (IGH), Centre National de la Recherche Scientifique (CNRS), Université de Montpellier (UM), 34000 Montpellier, France; sofia.kossida@igh.cnrs.fr

**Keywords:** Notch family members, neurodegenerative diseases, CADASIL, genetics, polymorphism analysis, mutation analysis, EGF, cysteine, protein structure analysis

## Abstract

The evolutionary conserved Notch signaling pathway functions as a mediator of direct cell–cell communication between neighboring cells during development. Notch plays a crucial role in various fundamental biological processes in a wide range of tissues. Accordingly, the aberrant signaling of this pathway underlies multiple genetic pathologies such as developmental syndromes, congenital disorders, neurodegenerative diseases, and cancer. Over the last two decades, significant data have shown that the Notch signaling pathway displays a significant function in the mature brains of vertebrates and invertebrates beyond neuronal development and specification during embryonic development. Neuronal connection, synaptic plasticity, learning, and memory appear to be regulated by this pathway. Specific mutations in human Notch family proteins have been linked to several neurodegenerative diseases including Alzheimer’s disease, CADASIL, and ischemic injury. Neurodegenerative diseases are incurable disorders of the central nervous system that cause the progressive degeneration and/or death of brain nerve cells, affecting both mental function and movement (ataxia). There is currently a lot of study being conducted to better understand the molecular mechanisms by which Notch plays an essential role in the mature brain. In this study, an in silico analysis of polymorphisms and mutations in human Notch family members that lead to neurodegenerative diseases was performed in order to investigate the correlations among Notch family proteins and neurodegenerative diseases. Particular emphasis was placed on the study of mutations in the Notch3 protein and the structure analysis of the mutant Notch3 protein that leads to the manifestation of the CADASIL syndrome in order to spot possible conserved mutations and interpret the effect of these mutations in the Notch3 protein structure. Conserved mutations of cysteine residues may be candidate pharmacological targets for the potential therapy of CADASIL syndrome.

## 1. Introduction

Research on *Drosophila melanogaster* with notched wings led to the discovery of the Notch gene in 1914 [1]. To date, it seems that the evolutionary history of the Notch family is closely related to the biological tree of life. The Notch protein and its homologs, Notch1, Notch2, Notch3, Notch4, LIN-12, and GPL-1, have been detected in the genomes of all kingdoms, demonstrating the evolutionary development of the Notch family [2]. Members of the Notch family were discovered to have a comparable structure across several kingdoms, extending from bacteria to chordates [3]. Only one Notch receptor is found in *D. melanogaster*. The Notch receptors LIN-12 and GLP-14 in *Caenorhabditis elegans* are redundant [4]. Mammals have four Notch paralogs, Notch1, Notch2, Notch3, and Notch4, displaying both redundant and distinct activities [5].

The Notch receptors (Notch1–Notch4), found in mammalian cells, are four different transmembrane proteins expressed on the cell’s surface as heterodimers not covalently bonded [6]. Notch proteins have an extracellular domain (NECD) that operates as the signal receiver and a transmembrane–intracellular domain (NICD) that operates as the signal transducer. The Notch1–Notch4 ECDs contain 36, 35, 34, and 29 epidermal growth factor-like repeats (EGF-like domain), respectively. Also, the ECD of Notch receptors has three cysteine-rich Lin12-Notch repeats (LNRs) and a heterodimerization domain (HD). The Notch ICD has an RBPJk-associated molecule domain (RAM) and nuclear localization sequences (NLSs) on both sides of the ANK domains. Also, Notch ICD consists of five to six ankyrin repeats (ANK), a transcriptional activation domain (TAD), and a C-terminal domain (PEST) rich in proline, glutamic acid, serine, and threonine. Notch family proteins function as cell surface receptors and direct regulators of gene transcription, constituting a particular signal transduction pathway that enables cells to affect the gene expression of their neighboring cells [7]. Notch signaling is activated upon cell-to-cell contact due to interactions between four transmembrane receptors encoded by Notch genes (Notch1–4) and five Notch ligands encoded by JAG1, JAG2, and DLL1, DLL3, and DLL4 [8]. Notch ECD contains EGF-like repeats that condense ligand–receptor binding [9]. 

The human (*Homo sapiens*) Notch1 gene is found at locus 9q34.3 on chromosome 9. Loss of function of the Notch1 protein is linked to abnormalities in angiogenesis, cardiogenesis, and somitogenesis, which could lead to the death of an embryo. This gene is involved in forming the first definitive adult hematopoietic stem cells (HSCs) [10]. Moreover, the development of B and T cells is regulated by Notch1 signaling. Mutations in the signaling and transcriptional regulator Notch1 result in various developmental aortic valve abnormalities, severe valve calcification, and T-cell acute lymphoblastic leukemia [10,11]. The Notch2 gene is located on chromosome 1p12. Notch2 has specific functional activity in determining cell fate and in the development of kidney, ovary, smooth muscle, T, and B cells [12]. Postnatal signaling regulates homeostasis, bone regeneration, and immune system function [13,14]. Mutations resulting in excessive Notch2 activity may lead in systemic issues typical of Alagille and Hajdu–Cheney syndromes such as heart abnormalities, chronic cholestasis, osteoporosis, polycystic kidneys, skeletal deformities, and neurological disorders [15,16]. The Notch3 gene is found between locations 13.2 and 13.1 on the short arm (p) of chromosome 19. This large type I transmembrane receptor, mostly expressed in pericytes and vascular smooth muscle cells adjacent to the local blood arteries, takes part in maintaining and renewing tissues as well as in important developmental functions [17]. Overexpression and aberrant activation of the Notch3 gene are linked to cancer, particularly breast and ovarian cancer. Mutations in Notch3 have been directly linked to the CADASIL syndrome [18]. The Notch4 gene is found at locus 6p21.32 on chromosome 6. It has been observed that both the overexpression and mutations of the Notch4 gene are related to cancer [19]. Notch4 is considered a new biomarker of cancer stem cells (CSCs) [20].

Notch genes are involved in various critical biological processes including somitogenesis, angiogenesis, vasculogenesis, cardiac development and function, neuronal development, and the specification and maintenance of neural stem cells (NSCs) [21]. All four mammalian Notch receptor paralogs and several pathway components (ligands and targets) are expressed with different cell type specificities in both the adult mouse and human brain [22]. There is evidence of Notch receptor expression in neurons (Notch1 and Notch2), neural stem cells (Notch1 and Notch2), vascular smooth muscle cells and pericytes (Notch3), endothelial cells (Notch1 and Notch4), and astrocytes (Notch1 and Notch2) [22]. Notch has been linked to maintaining NSCs in an undifferentiated state, preventing neuronal development, and even causing terminal differentiation inside the astrocyte lineage [23,24]. Notch signaling is essential for neural stem cell maintenance and neurogenesis in both the embryonic and adult brains [6]. The elderly’s brain function, cell differentiation, and neurite formation are all impacted by Notch signaling, which is crucial for the nervous system’s regular operation [5,25]. Accordingly, multiple mutations in Notch proteins have been linked to neurodegenerative conditions [21].

Quantitative data on this pathway’s structural, biochemical, and biophysical features have emerged during the last few years [26]. Various loss-of-function mutations in the embryo and adult highlight the critical role of Notch signaling. Numerous studies on neurogenesis have used *Drosophila melanogaster*, zebrafish, and mice as model species [6]. The conditional loss of Notch signaling in the embryo causes the precocious differentiation of NSCs and neurodevelopmental defects such as impaired survival and the aberrant migration of progenitor cells [6]. In the adult brain, NSCs are predominantly quiescent and rarely divide. However, it is likely that quiescent NSCs enter the cell cycle and transform into active NSCs before quitting the cell cycle again and reentering the quiescent state [27]. In adults, Notch signaling pathway mutations are involved in many neurodegenerative diseases and brain disorders [6].

Neurodegenerative diseases are incurable disorders of the central nervous system that present clinically and pathologically in various ways and damage particular neuronal subsets and anatomical functioning systems [28]. There are currently no therapies that target the underlying cause of neurodegenerative diseases. Therefore, it is not feasible to prevent or stop the progression of these disorders [29]. The involvement of Notch receptor genes and proteins in aging, cerebrovascular disorder, and Alzheimer’s disease is significant. Notch signaling may be a fundamental overlap between age-related vascular and Alzheimer’s pathogenesis that contributes to their comorbidity and combined impact on cognitive decline and dementia. Numerous results from genetics, cell culture model studies, and neuropathology all point to a connection between aberrant Notch signaling and the pathogenesis of Alzheimer’s disease [21]. In addition, it is generally established that the Notch3 protein plays a significant role in the development of CADASIL [30,31,32].

Cerebral Autosomal Dominant Arteriopathy with Subcortical Infarcts and Leukoencephalopathy is a hereditary dominant rare disease caused by mutations in the Notch3 protein, affecting adults beyond middle age and resulting in dementia and disability [33]. CADASIL is a fatal late-onset disease that primarily appears as a degenerative disorder of the central nervous system, and it is defined by specific clinical, neuroradiological, and pathological characteristics [30,34]. Over the last two decades, extensive efforts have been directed toward research on Notch3, identifying more than 280 mutations [35]. Some of these mutations cause a phenotype whereas others remain silent. Extensive analysis for categorizing, organizing, and mapping these mutations is required for a simple genotype–phenotype linkage [33]. Numerous pathogenic mutations in the Notch3 gene change the number of cysteine residues in the receptor’s extracellular domain, leading to protein misfolding and receptor aggregation [33]. Each EGF-like repeat contains six cysteines, which combine to create three disulfide bonds and provide the EGF repeat its three-dimensional structure [36]. However, non-Cys mutations have also been reported in recent years. These mutations do not match the disease’s typical pattern and pathology [30]. Even though most of the mutations in Notch3 are point mutations, it has been established that each one has a major impact on the three-dimensional structure of the Notch3 protein [30].

**Figure 1:**
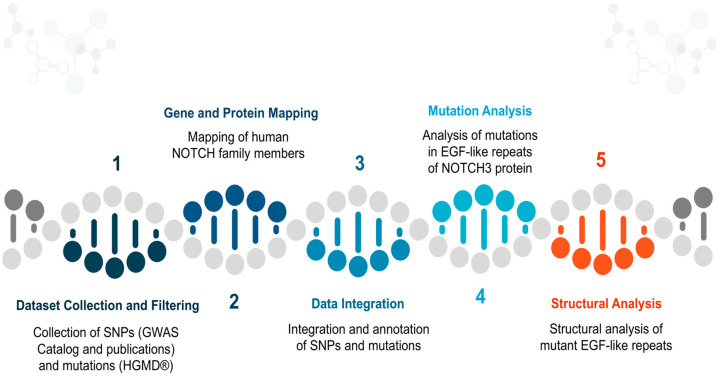
Flow chart presentation of the bioinformatic method, presented in five steps.

The study of the Notch family has increased, significantly, the availability of biological data on polymorphisms and mutations that are related with neurodegenerative diseases [15,21,37,38,39]. The initial purpose of this work was to gather all the cases and link them between nucleotides and protein sequences. Today, the scientific research seems to be focused on understanding the way the EGF region functions due to the significant mutagenesis it presents through a series of scientific publications [39,40,41,42,43]. The ultimate goal of this study is the holistic study of all mutations occurring in *Notch3*, with an emphasis on the EGF region and the CADASIL syndrome, in order to identify specific patterns of mutagenesis in the EGF-repeats that may be related to the clinical phenotype, sex, and age data of the various patients [38,39,44,45,46]. The study and analysis of all mutations can additionally open new horizons thus contributing to the identification of new pharmacological targets as well as contributing to the identification of a candidate treatment against CADASIL syndrome and, generally, neurodegenerative diseases. The outline of the integrated bioinformatic method is presented in Figure 1.

## 2. Materials and Methods

### 2.1. Dataset Collection and Filtering

Data were collected from polymorphism databases, disease-specific mutation databases, and publications. Specifically, single-nucleotide polymorphisms (SNPs) on Notch1–Notch4 genes associated with neurodegenerative diseases were extracted from available online databases such as GWAS-Catalog, dbSNP, LitVar, and ClinVar. Likewise, a second search was carried out in the online database PubMed (https://pubmed.ncbi.nlm.nih.gov/, accessed on 18 March 2024) for publications that contained the key terms “Neurodegenerative diseases”, “Cognitive Disorders”, “Alzheimer’s disease”, “CADASIL” AND “NOTCH1”, “NOTCH2”, “NOTCH3”, and “NOTCH4” with no date restriction. The collected SNPs from all databases were extracted, filtered, and annotated using Matlab bioinformatics toolbox for data mining and semantic techniques. All SNPs causing mutations on the protein level and directly related to neurodegenerative diseases were stored the final dataset. The Human Gene Mutation Database (HGMD^®^) (https://www.hgmd.cf.ac.uk/ac/index.php, accessed on 18 March 2024) was searched for missense mutations on Notch1–Notch4 proteins. HGMD^®®^ attempts to aggregate all known (published) human gene mutations responsible for human diseases. The mutations associated with neurodegenerative diseases have been collected. For each mutation, the access number, codon change, mutation position, and the phenotype it induces were recorded.

### 2.2. Gene and Protein Mapping

In this step, mapping of human Notch family genes and proteins was accomplished. The terms “Notch1”, “Notch2”, “Notch3”, and “Notch4” were searched on the NCBI database (https://www.ncbi.nlm.nih.gov, accessed on 18 March 2024) while the filter “Gene” was previously selected for extracting the nucleotide sequences of these genes. Furthermore, additional information like gene location, chromosome, nucleotide sequence length, access number, and alternative gene names was extracted from the NCBI database. A second search was carried out on NCBI database with the “protein” filter for extracting the amino acid sequences of human Notch family proteins. These terms were also searched on available online protein databases such as UniProt and InterPro. Information on the amino acid sequence lengths of proteins and disease involvement was obtained. Domains of each protein were also recorded. Protein domain data were also extracted from publications in the PubMed database.

### 2.3. Data Integration

The data collected from polymorphism databases and mutation databases were merged and annotated. SNPs and the mutations in the human Notch family associated with neurodegenerative diseases were integrated to correlate polymorphisms and mutations. The integrated data are presented in a table, providing information for SNP ID, nucleotide change, codon change, mutation position, domain in which the mutation is located, and the phenotype it causes. The finding of correlations among polymorphisms and mutations on Notch1–Notch4 was necessary to find out which ones and how many mutations are associated with a known polymorphism, which neurodegenerative disease is most often caused by Notch3 mutations, which Notch3 domain contains the majority of these mutations, and which amino acid appears to be most frequently mutated. To comment on these queries, specific diagrams have been created where the data analysis is presented.

### 2.4. Mutation Analysis

The majority of polymorphisms/mutations associated with neurodegeneration were located in Notch3 and specifically in the EGF region. For this reason, our work then focused on this domain (Table 7). In order to analyze the mutations identified in the EGF-like repeats of the Notch3 protein, a FASTA file with the amino acid sequences of 34 EGF-like repeats was created. This file was analyzed using the Jalview platform (https://www.jalview.org/, accessed on 18 March 2024). JalView computes and visualizes a large number of sequences with high performance. The main advantage of this method is that it allows for the identification of conserved motifs with a quick overview of alignment. In this step, a multiple alignment of 34 EGF-like amino acid sequences was performed in order to find the conserved amino acids in the EGF-like repeats. Jalview’s comments section, which displays amino acid conservation with logos and histograms, was also examined to discover novel motifs. The sequencing results were further elaborated. A histogram was created, showing the percentage of mutations at each amino acid position in the EGF-like sequences according to multiple-sequence alignment numbering. Finally, mutations in conserved amino acids in the EGF-like repeats were studied to identify conserved amino acid changes. A chart presenting the results of this analysis was also constructed.

### 2.5. Structural Analysis

Using the MOE (Molecular Operating Environment) platform, the mutant EGF-like repeat structure was analyzed to figure out the consequences for the Notch3 protein when including amino acid changes. MOE 2019.01 is an integrated life sciences software that supports drug design through molecular simulation, protein structure analysis, small molecule processing data, protein binding, and small molecule design. A homology modeling of the Notch3 approach was used for the structural analysis since no structure has been determined for this protein. The homology modeling of Notch3 protein structure was extracted from the AlphaFold Protein structure Database (https://alphafold.ebi.ac.uk/, accessed on 18 March 2024). Structural analysis of the EGF-like 2 repeat was performed while introducing conserved mutations associated with CADASIL syndrome.

## 3. Results

### 3.1. Dataset

As a consequence of systematic data mining, SNPs of human Notch family members correlated with neurodegenerative diseases were identified (Table 1). These polymorphisms have been derived both from the data mined from the biological database GWAS CATALOG as well as from the publications that contained the ontologies of interest based on PubMed searches. In total, 1887 relevant publications were extracted, of which 188 described polymorphisms. Only single-nucleotide polymorphisms that cause mutations at the protein level and are directly related to neurodegenerative diseases were used. In the Notch1 gene, 57 polymorphisms were collected from online polymorphism databases and publications, of which 41 missense variants were screened. Only one variant was identified in this gene related to Alzheimer’s disease (Table 4). Twenty-six polymorphisms in the Notch2 gene were retrieved. Among these, 15 missense SNPs were examined, and only one was found to be associated with autism multiplex disorder (Table 5). A total of 59 polymorphisms in the Notch3 gene were extracted from online databases and publications. Twenty-eight missense SNPs were screened, and all were found to be involved in the manifestation of neurodegenerative diseases (Table 7). Most SNPs are associated with CADASIL disease. Finally, from the Notch4 gene were extracted only two SNPs (missense variants), and neither was found to be associated with neurodegenerative disease (Table 6). Unlike SNPs in Notch1, Notch2, and Notch4, missense SNPs in the Notch3 gene seem more strongly related to neurodegenerative disorders. Additionally, through filtering the results of the HGMD database, all mutations in Notch1–Notch4 associated with neurodegenerative diseases were reported and imported. More specifically, 1, 1, 312, and 4 mutations in Notch1, Notch2, Notch3, and Notch4, respectively, were identified to be correlated with neurodegenerative diseases.

### 3.2. Gene and Protein Mapping

Data on Notch1–Notch4 genes and proteins were retrieved from the National Center for Biotechnology Information (NCBI), UniProt, and InterPro. NCBI-Gene results for Notch1–Notch4 are shown in Table 2. The search results for Notch1–Notch4 proteins are shown in Table 3. Notch1 has the greatest amino acid sequence length of the four Notch proteins (2555 aa), followed by Notch2 (2471 aa), Notch3 (2321 aa), and Notch4 (2003 aa). Mutations in these genes are linked to the manifestation of several diseases. Mutations in the Notch1 gene are associated with diseases such as aortic valve disease, type 1 (AOVD1), Adams–Oliver syndrome type 5 (AOS5), T-cell acute lymphoblastic leukemia (T-ALL), chronic lymphocytic leukemia, and squamous cell carcinoma of the head and neck. Mutations in Notch2 are linked to Hajdu–Cheney syndrome, Alagille syndrome 2 (ALGS2), and cancer. Mutations in Notch3 have been identified as the cause of diseases such as CADASIL, infantile myofibromatosis, early-onset arteriopathy with cavitating leukodystrophy, lateral meningocele syndrome, and cancer. Finally, mutations in the Notch4 gene may be associated with schizophrenia.

**Figure 2:**
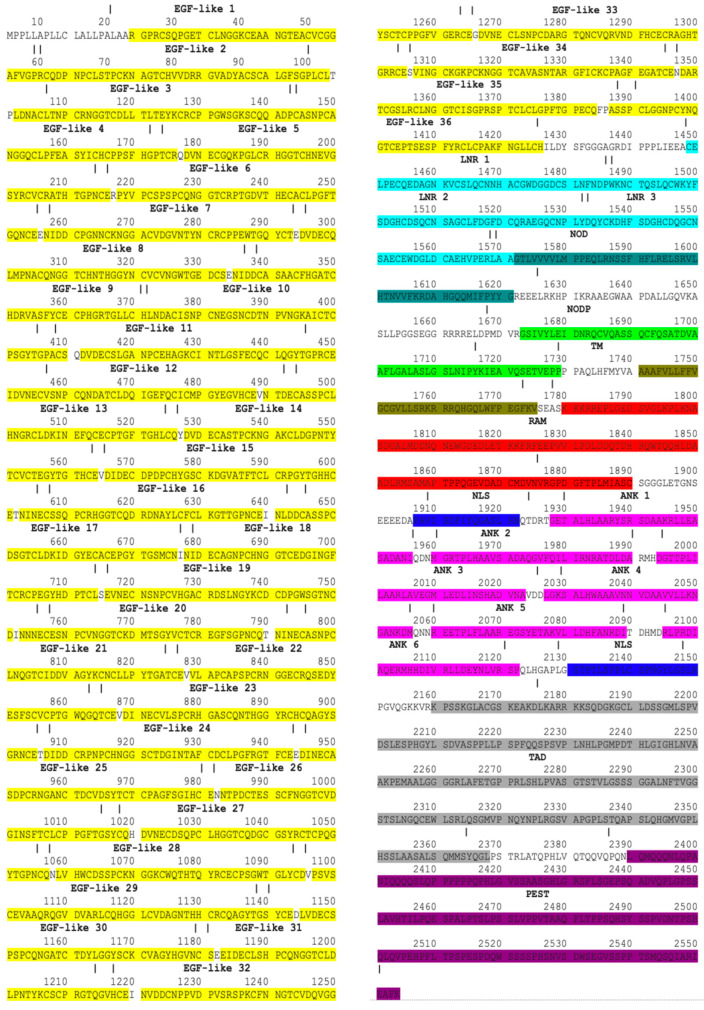
Notch1 protein domains. Colors represent protein domains.

**Figure 3:**
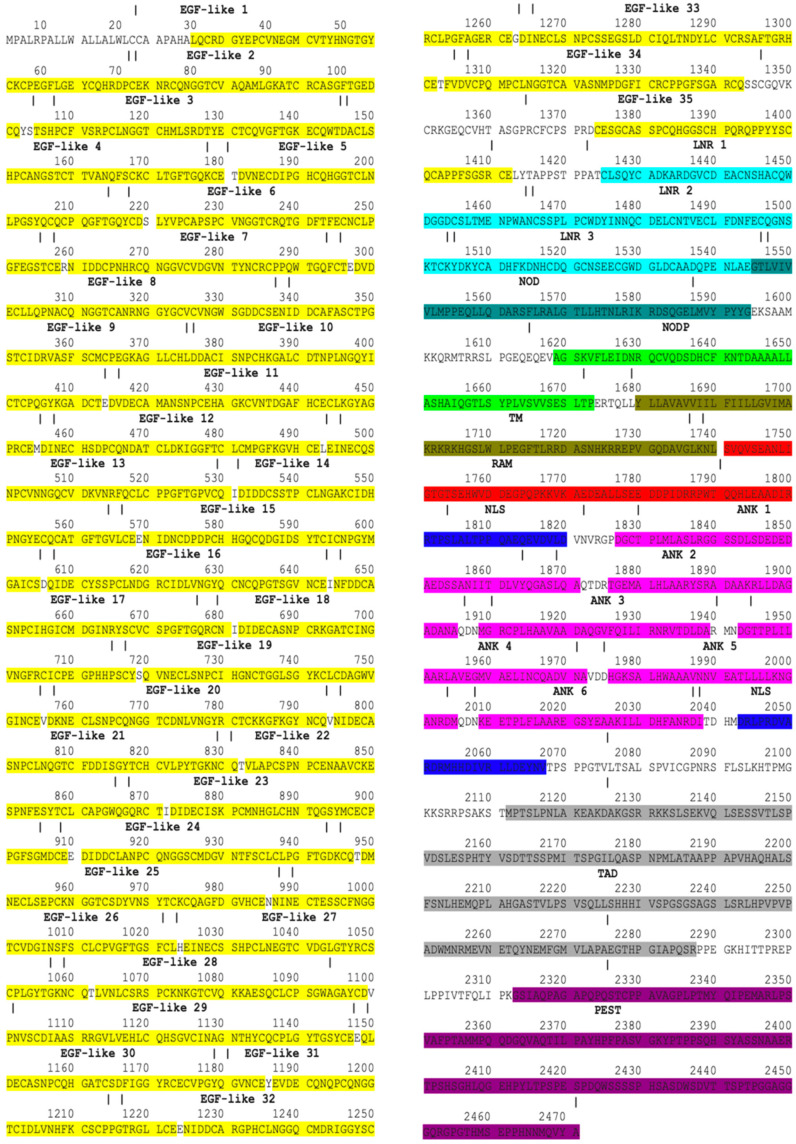
Notch2 protein domains. Colors represent protein domains.

**Figure 4:**
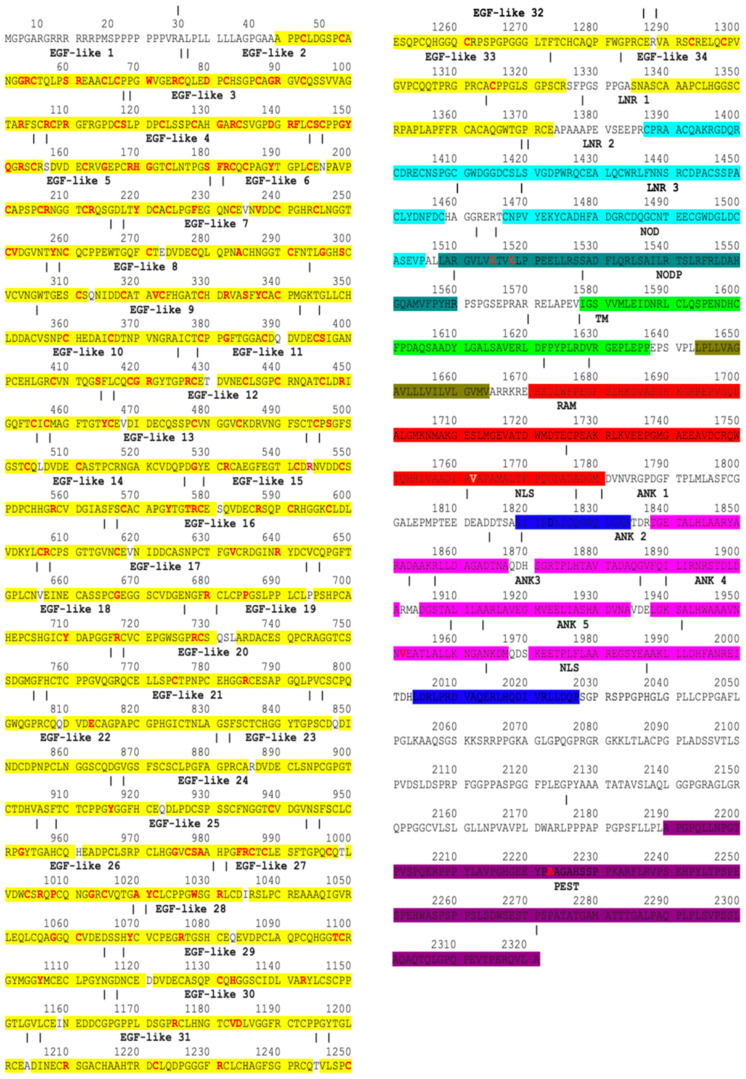
Notch3 protein domains. Amino acids marked with bold red represent the CADASIL mutations. Colors represent protein domains.

**Figure 5:**
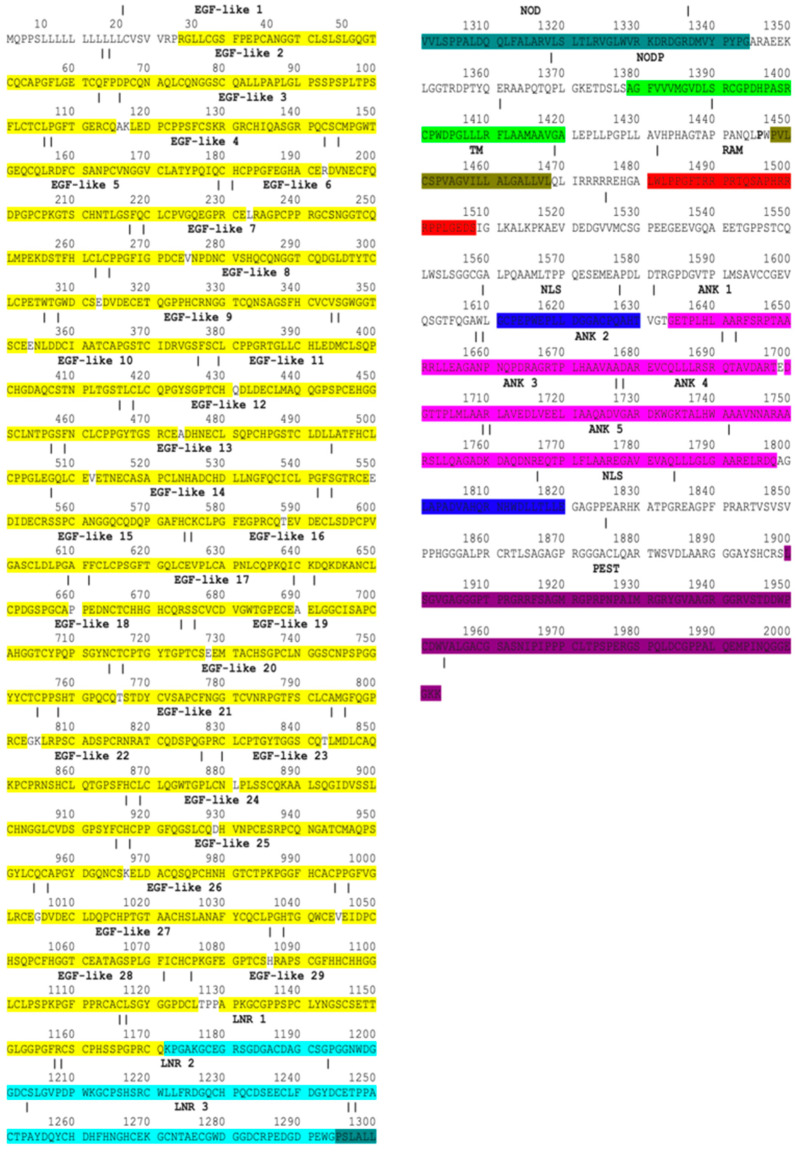
Notch4 protein domains. Colors represent protein domains.

By mining information from databases and publications, Notch1–Notch4 proteins were mapped. The Notch1–4 proteins consist of EGF, LNR, NOD, NODP, TM, RAM, NLS, ANK, TAD, and PEST domains [47]. In mammals, the TAD region is present in Notch1 and 2 but not in Notch3 and 4 [48]. The EGF domain is made up of EGF-like repeats. In human Notch1, Notch2, Notch3, and Notch4, the EGF domains consist of 36 EGF-like, 35 EGF-like, 34 EGF-like, and 29 EGF-like repeats, respectively. Each EGF-like repeat comprises 30–40 amino acids and contains six cysteine residues (C). The LNR sector consists of three micro-domains, LNR1, LNR2, and LNR3. The ANK domain in Notch1–2 proteins is made of six ankyrin repeats while in Notch3-4, proteins are made of five ankyrin repeats. Notch1–Notch4 protein domains are demonstrated with specific colors in protein sequences in Figure 2, Figure 3, Figure 4 and Figure 5. 

### 3.3. Data Integration

**Figure 6:**
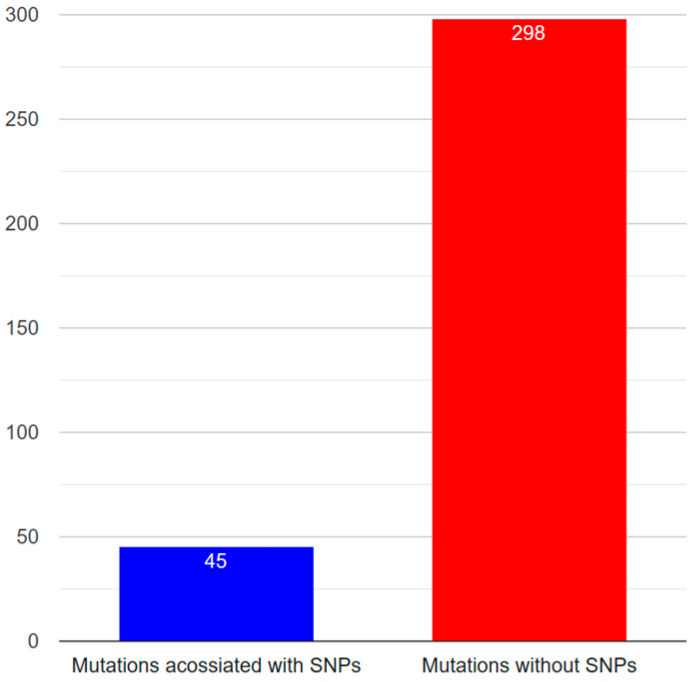
Number of associated and unassociated mutations with known SNPs.

The integrations of polymorphism and mutation datasets of Notch1–Notch4 are shown in Table 4, Table 5, Table 6 and Table 7. Since Notch3 is associated with the manifestation of neurodegenerative diseases to a considerably more significant degree than other human Notch family members, the annotation of Notch3 data followed. In Table 6, the consolidated data from the recording of Notch3 polymorphisms and mutations are presented in order to correlate them. Information on nucleotide change, amino acid change, protein domain on which the mutation is located, and the phenotype it induces were obtained for the recorded SNPs. According to the association between polymorphisms and mutations reported, 23 polymorphisms are related to mutations not identified in the HGMD data source. A blank cell in the “Accession number” column of the table indicates the particular polymorphisms. Although there are 43 mutations associated with known SNPs, there are 292 mutations unrelated to any identified SNP (Figure 6).

**Figure 7:**
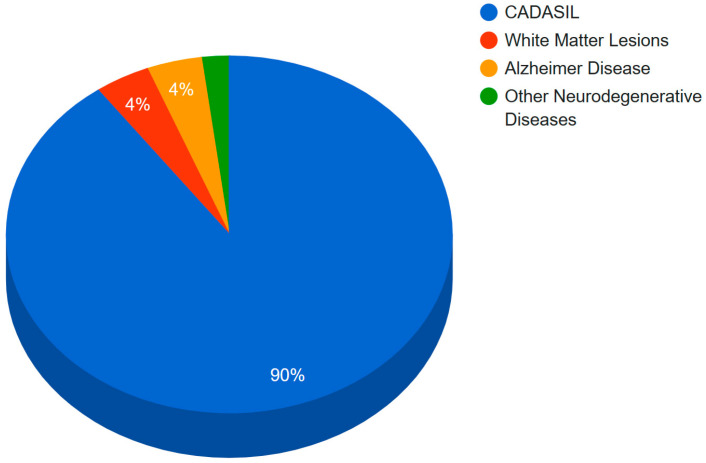
Percentage of Notch3 mutations associated with a specific neurodegenerative disease.

The percentage of mutations in Notch3 associated with neurodegenerative diseases is displayed graphically in a pie chart in Figure 7. According to this study, 90% of Notch3 mutations lead to CADASIL disease, 4% of Notch3 mutations lead to Alzheimer’s disease, and 4% of Notch3 mutations lead to white matter lesions. Only 2% of Notch3 mutations are associated with other neurodegenerative diseases such as the small-vessel disease of the brain, ischemic stroke, migraine, and autism. Since CADASIL is represented by 90% of mutations in Notch3, a guide map (Figure 4) was created for Notch3 mutations. Mutations in the amino acid sequence of the Notch3 protein are marked in bold red. 

**Figure 8:**
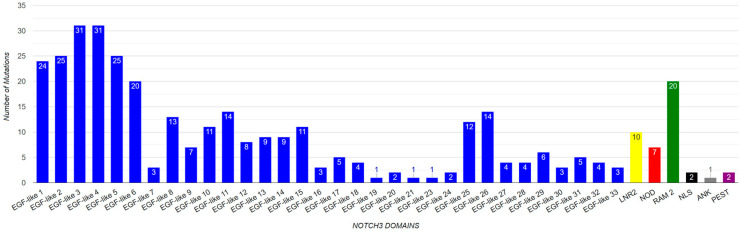
Demonstration of mutation number per Notch3 domain.

**Figure 9:**
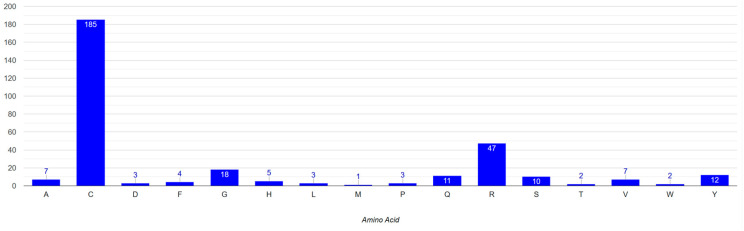
Mutated amino acid residues in Notch3 protein associated with CADASIL.

There have been reported to be 310 mutations in Notch3 that cause CADASIL syndrome (Table 7). The majority of the mutations (305), as shown in the amino acid sequence of Figure 4, are found in the EGF domain. The distribution of mutations in the EGF-like repeats and other protein domains is illustrated through a chart (Figure 8). The highest concentration of mutations is observed in the EGF-like 3 and EGF-like 4 repeats while the lowest numbers of mutations are found in the NOD, RAM, and PEST domains. In addition, more than 60% of Notch3 protein mutations that lead to CADASIL disease occur at the cysteine residue (Figure 9).

### 3.4. Mutation Analysis

**Figure 10:**
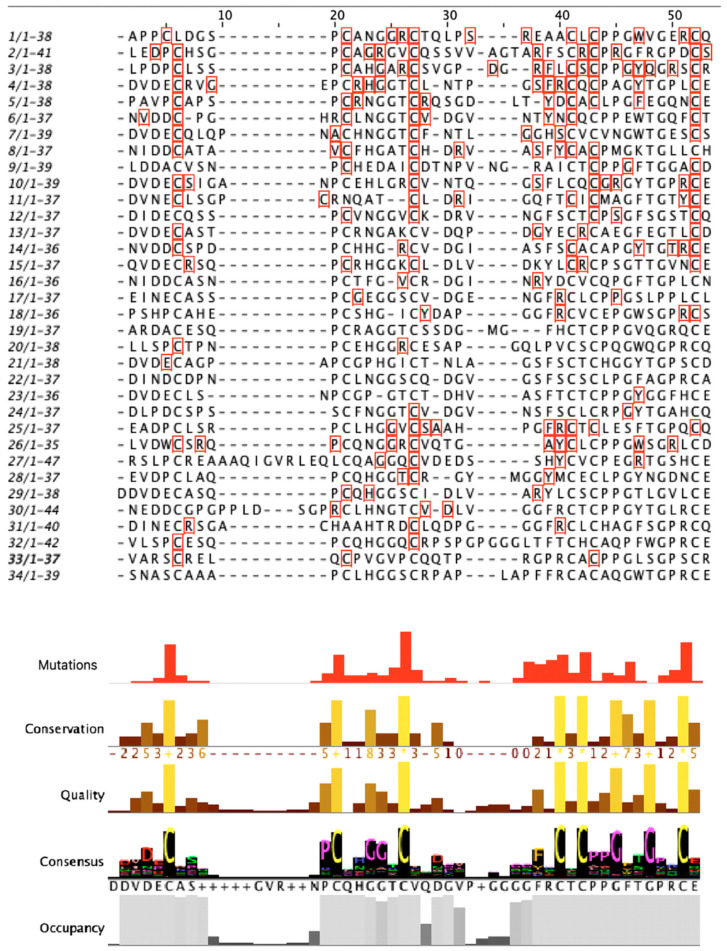
Conserved amino acids based on the sequence alignment of the EGF-like repeats. Each EGF-like repeat is presented with the specific number and the sequence length. The amino acids marked with the red square are the mutated ones.

Since 90% of the mutations identified in *Notch3* and related to neurodegenerative diseases were located in the EGF region, the mutation analysis was mainly focused on this specific region. The multiple-sequence alignment (MSA) of amino acid sequences of Notch3 EGF-like repeats was performed to identify highly conserved amino acids within 34 EGF-like repeats. EGF-like repeats have a significant role in Notch signaling [2]. Six cysteine residues in each EGF repeat generate disulfide bonds affecting their native three-dimensional structure. Consequently, they are a crucial component of the EGF domain, and mutations in these residues lead to a pathological phenotype, specifically in CADASIL syndrome [2,30,49]. As shown by the present multiple alignment in the visualized results of the histogram “conservation” (Figure 10), cysteine residues are conserved within all EGF-like repeats. More particularly, the “consensus” histogram (Figure 8) shows the percentage of conserved amino acids at each position. Based on the data, cysteine residues are 100% conserved at positions 27, 41, 43, and 52 while positions 6 and 21 are 97% and 94% conserved, respectively. Additionally, glycine is conserved at positions 49 (97%), 46 (91%), 25 (71%), and 24 (76%), and proline is conserved at position 20 (74%). The greatest concentration of mutations appears at the cysteine residues. Almost 60% of cysteines at position 27 and 50% at positions 6 and 52 were identified as mutated. Results for the identified mutation percentage in each amino acid position of the EGF sequences are demonstrated in the histogram “mutations” (Figure 10). Most of the mutations are identified in positions 6, 27, and 52 of EGF-like repeats. 

**Figure 11:**
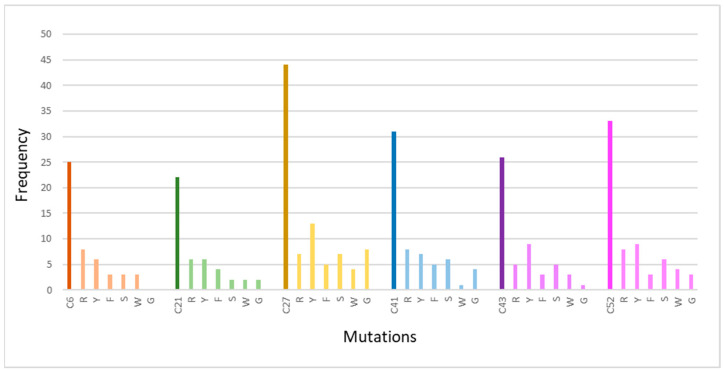
Presentation of cysteine mutations at positions (C6, C21, C27, C41, C43, and C52) based on the frequency of occurrence of a specific mutation. Τhe chart’s bold-colored columns represent the total number of conserved cysteine mutations of 34 EGF-like repeats.

The cysteine residues with the greatest level of conservation were analyzed to determine how frequently a certain amino acid change occurs at these positions (Table 8). The conserved cysteine residues are C21, C27, C41, C43, and C52. The frequency of each mutation is represented graphically in Figure 11. Based on the genetic background, more particularly based on the triplets that code for the amino acids replacing the cysteine, the appearance of the specific mutations was expected. The changing of one nucleotide in the triplets coding for cysteine (TGT, TGC) leads to the coding of another amino acid that has two common nucleotides with cysteine. Despite the fact that all cases of cysteine mutations associated with CADASIL syndrome have been reported (Table 8), it is vital to note that no nonsense mutation (TGA) has been reported as a cause of CADASIL. Although, based on the genetic code, the specific mutations are expected, their different frequencies of occurrence lead to the conclusion that these mutations are also related to genetic drift. Most frequently, cysteines were found to be mutated into arginine and tyrosine. This analysis also revealed that cysteines C27S/R/Y/W/F (EGF 5), C43S/R/Y/W/F (EGF 2), and C52S/R/G/F/Y (EGF 4) appeared to be more sensitive to pathogenic changes.

### 3.5. Structural Analysis

**Figure 12:**
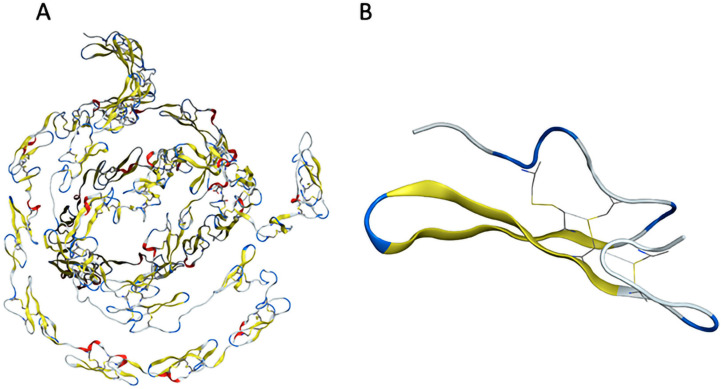
Structural representation of the EGF domain and the EGF-like 2 repeat of the Notch3 protein. (**A**) Structure of EGF domain. (**B**) Structure of EGF-like 2 wild-type repeat.

The structural analysis of the mutations made it possible to understand the implications of inserting specific mutations into the amino acid sequence of the Notch3 EGF domain. Each EGF-like repeat consists of a set of two anti-parallel β-sheets (Figure 12). The structural stability of the Notch3 protein is maintained via disulfide bridges established by a set of strategically positioned cysteine residues [50]. As the key element of the domain, the six cysteine residues of the EGF-like repeat are crucial for the creation of disulfide bonds determining the native 3D structure of Notch proteins (Figure 12B).

**Figure 13:**
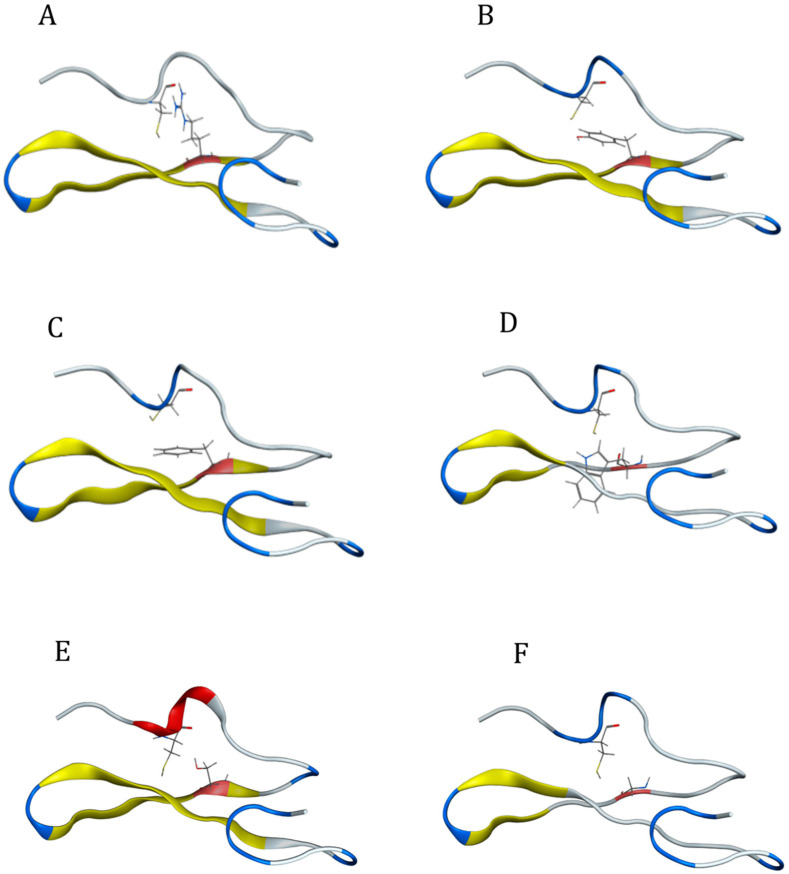
Structural representation of the mutated EGF2 repeat of the Notch3 protein in the cases where the cysteine at position 27 mutated: (**A**) C27R, (**B**) C27Y, (**C**) C27F, (**D**) C27W, (**E**) C27S, and (**F**) C27G. In the EGF structure, the anti-parallel β-sheets are shown in yellow and the location of the mutated cysteine is shown in orange.

Mutations in the cysteine residue at position 27 of EGF2 partly rearranged and destabilized the structure of EGF-like repeat due to the destruction of the disulfide bridge between the mutant cysteine and another cysteine residue (Figure 13). A change in the structure of the EGF 2 repeat was induced in each case of cysteine mutation.

Cysteine residue has a polar, uncharged side chain. The thiol group imparts polarity to cysteine. The induced C27R mutation leads to a broken disulfide bond between the mutant cysteine in one of the β sheets and its interacting cysteine in the coil opposite the β-sheet (Figure 13A). Arginine is a positively charged amino acid with a long side chain that carries a charged guanidine group. The full positive charge of the arginine side chain interacted with the partial negative charge of the cysteine side chain in this study. Even though there was no change in the β-sheet structure, this mutation seemed to affect the coil where the cysteine is located, causing the expansion of the coil because arginine is larger than cysteine. Thus, with the opening of the peptide chain, the space that could accommodate the arginine was created, causing the disturbance of the EGF-like 2 structures. 

The C27Y and C27F mutations did not significantly change the structure of EGF despite breaking the disulfide bond. The C27Y mutation results in the differentiation of the coil opposite to tyrosine since tyrosine is larger than cysteine (Figure 13B). Tyrosine is a non-polar amino acid that contains an aromatic side chain. Due to the presence of the hydroxyl group in the side chain, tyrosine is predicted to interact via hydrogen bonding with cysteine. C27F mutation leads to a similar differentiation of the EGF 2 coil since phenylalanine is also an aromatic amino acid with a larger side chain than cysteine (Figure 13C). Phenylalanine is a non-polar amino acid and does not interact with cysteine.

The C27W mutation destroys the disulfide bond and causes a partial loss of the structure of both anti-parallel β sheets (Figure 13D). The large, conjugated side chain of tryptophan got away from the EGF2 core. Due to the lack of available space, 27W was expected to be outside the β-sheet structure. On the contrary, it was found in the available space at the same level defined by the two β-sheets. As a result, the two β-sheets lost their original form as pleated surfaces and were converted into a coil structure.

The C27S mutation destroyed a disulfide bond in the EGF-like 2 structure (Figure 13E). This led to the rearrangement of the coil structure where the cysteine, which interacts with the serine, is located. Dipolar–dipolar interactions between serine and cysteine may have caused this rearrangement. Consequently, an α helix is formed in this region of the EGF2 structure.

The C27G mutation destroys the disulfide bond and causes the partial loss of the two anti-parallel β-sheet structures (Figure 13F). Glycine is a non-polar amino acid that carries a non-polar aliphatic side chain. In the absence of a side group, there is no stereochemical barrier for glycine, allowing it to adopt a variety of conformations that could result in polypeptide chain curving and enhanced flexibility. This feature of glycine may also be responsible for the partial loss of β-sheets and rearrangement into coils.

## 4. Discussion

Decades of research have shown the significance of the Notch signaling pathway in neural development. More recent studies have proven that Notch receptors continue to be expressed and active in numerous areas of the adult central nervous system [51,52,53].

Adult neurogenesis, memory, synaptic plasticity, acute brain trauma, and chronic neurological diseases have all been linked to Notch signaling [22]. The analysis of mutation datasets revealed that human Notch1, Notch2, and Notch4 proteins are not significantly associated with neurodegenerative diseases [21]. On the other hand, most mutations in Notch3 lead to neurodegenerative diseases, mainly CADASIL syndrome [21,33]. Consequently, the current in silico study yielded new insights that might contribute to a better understanding of the correlation between neurodegenerative disorders and the human Notch family. 

Considerable focus was given to analyzing Notch3 protein mutations associated with CADASIL disease. The study of mutations in the Notch3 protein is crucial because it could contribute to a better understanding of the molecular mechanisms that cause the disease, which is easier to study due to its monogenic nature. Even though the majority of mutations are point mutations, the effect of each on the three-dimensional structure of the Notch3 protein is significant [30,54]. Clinical genetics databases, including disease-specific mutation databases and genotype-phenotype research, provide a large amount of data on bioinformatics. Nevertheless, there is a scientific gap in linking the data provided by disease mutation databases and polymorphism databases [55]. Developing a database that provides a unified mapping of nucleotide sequences, protein sequences, and their protein domains, as well as polymorphisms and mutations related to human diseases, may pose a challenge for computational biology.

To date, a series of pathogenic mutations in Notch3 affecting the number of cysteine residues in the receptor’s extracellular domain and resulting in protein misfolding and receptor aggregation have been identified [54]. Cysteine is the most active amino acid since it is involved in a wide range of biological functions [56]. Within extracellular proteins, cysteines are frequently involved in disulfide bridges in which pairs of cysteines are oxidized to create a covalent bond. Disulfide bonds’ primary function is to stabilize protein structures. Cysteine generally has no preference for substituting with any other amino acid [57]. The reported cysteine substitutions in Notch3 ECD that induce CADASIL disease are arginine, tyrosine, phenylalanine, serine, glycine, and tryptophane. Generally, the extremely varied functions that cysteines play in extracellular proteins explain the below preferences for substitution: Arg (-5), Gly (-6), Tyr (-4), Phe (-5), Trp (-5), and Ser (-5) [33,57]. 

In addition, it has been observed that most frequently, the mutations associated with CADASIL occur in the first two nucleotides and much less frequently in the third nucleotide of the triplet that codes for the cysteine amino acid. Mutations in the first and second nucleotide of the cysteine triplet in this study led to the replacement of cysteine with arginine and glycine, as well as with serine, phenylalanine, and tyrosine. Cysteine substitution to tryptophane was noticed when a mutation occurred in the third nucleotide of the triplet. This study suggested that the first and second nucleotides are sensitive to mutations whereas the third nucleotide appears more conserved. Based on the genetic code, the occurrence of specific mutations was expected [58]. Also, the different frequency of occurrence of each of these mutations is considered linked to the genetic draft, which slowly eliminates the variability that mutations cause, thereby achieving a steady state [59] (high frequency of cysteine mutating to tyrosine and arginine). Recording only one case of nonsense cysteine mutation in the Notch3 protein leads to two possible conclusions. There is the possibility that nonsense cysteine mutations lead to diseases, but no more cases of these mutations have been identified. It is also possible that cases of nonsense cysteine mutations have resulted in fetal death and have not been identified. Consequently, the only mutations identified are the ones that result in non-physiological protein function and therefore cause neurodegeneration. The frequent occurrence of mutations in cysteine residues that are highly conserved in the EGF-like repeats of Notch3 leads to protein misfolding and the manifestation of CADASIL syndrome [54]. 

Based on the results of the present work, which stem from the specialized study of the EGF-like domain of Notch3, several beneficial conclusions emerge. The accumulation of mutations appears to be different between EGF-like repeats 1 and 34, and these mutations were significantly increased in key amino acids in each EGF-like repeat such as in cysteine, glycine, and arginine (Figure 9 and Figure 10). Today, with the increasing number of experimental data from patients with CADASIL syndrome, it is possible to create a mathematical model through which we will be able to relate the order and the series of mutations in different EGF-like repeats based on a specific phenotype of the disease, as well as based on sex and age [38,50,60,61]. Some studies also have made this observation [39]. In addition, based on the literature, we know the different phenotype in characteristics displayed by each patient that can perhaps be explained by the use of this mathematical model and the use of the above characteristics [61]. On the other hand, several attempts have been made to treat the disease based on the key amino acids that most mutations show in the EGF-like domain of Notch3 [33,40,60,62]. This particular work presents all the candidate positions as a holistic atlas in a detailed analysis of the changes both at the nucleotide and protein level for a contribution in this effort to fight CADASIL syndrome and neurodegenerative diseases in general [21,53].

## 5. Conclusions

To summarize, the present in silico study focused on analyzing mutations in Notch1–Notch4 proteins correlated with neurodegenerative diseases. The Notch pathway is crucial for the nervous system’s development and pathogenesis due to its strong association with stem/progenitor cell progression and extensive pleiotropy. Neurodegenerative diseases are conditions characterized by the progressive and slow degeneration of neurons resulting in aberrant cell function and cell death. So far, no therapies that cure or prevent the progression of neurodegenerative diseases by targeting their underlying causes have been developed. Current therapies for these disorders are limited to symptom treatment. The integration of molecular methods, such as nanomedicine, genomics, proteomics, bioinformatics, and the measurement of environmental toxic body burdens, holds great promise for accelerating the process of identifying specific risk factors and mechanisms of pathogenesis in order to develop effective therapies for these diseases. Due to their role in cell fate determination and cell communication, as well as the proteolytic process they undergo in the signaling pathway, Notch proteins could be used as promising therapeutic targets for neurodegenerative diseases. 

The ultimate aim of the in silico study was to uncover potential CADASIL disease-causing conserved mutations and analyze the consequences of these mutations in the protein structure. Based on the results obtained from the present work, the correlation of *Notch3* polymorphisms—mutations with neurodegenerative diseases, especially in CADASIL syndrome—are clearly evident. In particular, the results show the accumulation of most of them in the EGF region of the protein. This specific protein region appears to be very crucial in the biomolecule’s functionality, with changes in the EGF region appearing to lead to neurological pathologies. Through our analysis, we studied the contribution of specific sequence alterations, their frequency of occurrence at candidate sites in each EGF-like repeat, and their frequency of occurrence at specific key amino acids that appear to be conserved in each EGF-like repeat. In this direction, detailed molecular dynamics simulations showed that these conserved mutations trigger local rearrangements in the structure of the mutant EGF-like repeat of the Notch3 protein. The identified conserved mutations of cysteine residues could be used as supplementary pharmacological targets for the development of effective therapeutic schemes against CADASIL.

Since CADASIL syndrome is a monogenic disease, the opportunity to better interpret the mode of function of Notch proteins and their association with neurodegenerative diseases through mutations occurring in *Notch3* was utilized. Therefore, we propose the creation of a mathematical model through which we will be able to study, in detail, the importance and contribution of mutations in EGF-like repeats based on both their concentration, frequency of occurrence, and mutation pattern in each specific numbered EGF-like repeat as well as their detection in specific key positions described in this work. As it is known, EGF-like domains are prevalent in numerous protein families, suggesting that the employment of this specific mathematical model could potentially implicate both other proteins in neurodegenerative diseases as well as various other disorders. Furthermore, future objectives should encompass the comprehensive examination of the mutations delineated in this study, particularly those occurring within the intracellular domain, from both evolutionary and structural perspectives.

## Figures and Tables

**Table 1:** Dataset of collected SNPs and mutations from online databases.

	SNPs	Mutations	Neurodegenerative Diseases
Notch1	1	1	Mental retardation, autosomal dominant, Alzheimer’s disease
Notch2	1	1	Autism multiplex
Notch3	28	312	CADASIL, white matter lesions, Alzheimer’s disease, ischemic stroke, cerebral small-vessel disease, arteriopathy and cavitating leukoencephalopathy, autism, migraine
Notch4	0	4	Schizophrenia, multiple sclerosis, migraine

**Table 2:** NCBI data for *Notch1–4* genes.

Gene	Notch1	Notch2	Notch3	Notch4
Locus	9q34.3	1p12	19p13.12	6p21.32
DNA linear	58343 bp	165110 bp	48349 bp	36225 bp
Exon count	34	34	33	31
Accession number	NG_007458	NG_008163	NG_009819	NG_028190
Organism	*Homo sapiens*	*Homo sapiens*	*Homo sapiens*	*Homo sapiens*
Also known as	hN1; AOS5; TAN1; AOVD1	hN2; AGS2; HJCYS	IMF2; LMNS; CASIL; CADASIL; CADASIL1	INT3

**Table 3:** NCBI data for Notch1–Notch4 proteins.

Gene	Notch1	Notch2	Notch3	Notch4
Protein length	2555 aa	2471 aa	2321 aa	2003 aa
Accession number	NP_060087	NP_077719 XP_946791 XP_950472	NP_000426	NP_004548
Organism	*Homo sapiens*	*Homo sapiens*	*Homo sapiens*	*Homo sapiens*
Disease	T-cell acute lymphoblastic leukemia, Adams–Oliver syndrome, aortic valve disease, cancer	Hajdu–Cheney syndrome, Alagille syndrome, cancer	CADASIL, infantile myofibromatosis, early-onset arteriopathy with cavitating leukodystrophy, lateral meningocele syndrome, cancer	Schizophrenia

**Table 4:** SNPs and mutations in Notch1 associated with neurodegenerative diseases.

Notch1						
A/A	SNP ID	Nt Change NG_007458.1	Mutation	Domain	Phenotype	Accession Number
1	_	_	Y2116>TERM	TAD	Mental retardation, autosomal dominant	CM171556
2	rs79782048	36150G>A	Ε694>Κ	EGF-like 18	Alzheimer’s disease	

**Table 5:** SNPs and mutations in Notch2 associated with neurodegenerative diseases.

Notch2						
A/A	SNP ID	Nt Change NG_008163.1	Mutation	Domain	Phenotype	Accession Number
1	rs61752484	148130A>G	D1327G	EGF-like 34	autism multiplex	

**Table 6:** SNPs and mutations in Notch3 associated with neurodegenerative diseases.

Notch3							
A/A	SNP ID	nt Change NG_009819.1	Codon Change	Mutation	Domain	Phenotype	Accession Number
1	N/A		TGC-GGC	C43>G	EGF-like 1	CADASIL	CM052264
2	N/A		TGC-TCC	C43>S			CM1712096
3	N/A		TGC-TTC	C43>F			CM035648
4	N/A		TGT-CGT	C49>R			CM106868
5	N/A		TGT-GGT	C49>G			CM073243
6	rs193921045	8431G>A	TGT-TAT	C49>Y			CM971054
7	rs193921045	8431G>T	TGT-TTT	C49>F			CM052265
8	N/A		GGT-TGT	G53>C			CM106869
9	N/A		CGT-TGT	R54>C			CM003012
10	N/A		TGC-GGC	C55>G			CM1714276
11	N/A		TCC-TGC	S60>C			CM052266
12	N/A		CGG-TGG	R61>W			CM0910982
13	N/A		TGC-GGC	C65>G			CM108953
14	N/A		TGC-TAC	C65>Y			HM070086
15	N/A		TGC-TCC	C65>S			CM052267
16	N/A		TGC-AGC	C67>S			CM092086
17	N/A		TGC-TAC	C67>Y			CM034666
18	rs28937321	13478G>T	TGG-TGT	W71>C			
19	N/A		CGG-CCG	R75>Q			CM156963
20	N/A		CGG-CCG	R75>P			CM061880
21	N/A		TGT-CGT	C76>R			CM023649
22	N/A		TGT-TGG	C76>W			CM052268
23	N/A		TGT-TAT	C76>Y			CM1615079
24	N/A		GAC-GGC	D80>G	EGF-like 2	CADASIL	CM150655
25	N/A		TGT-CGT	C82>R			CM148255
26	N/A		TGT-CGT	C87>R			CM052269
27	N/A		TGT-TAT	C87>Y			CM044913
28	N/A		TGT-TTT	C87>F			CM1714277
29	N/A		GGC-TGC	G89>C			CM128781
30	N/A		CGT-TGT	R90>C			CM971055
31	N/A		TGC-GGC	C93>G			CM1714278
32	N/A		TGC-TAC	C93>Y			CM023650
33	N/A		TGC-TTC	C93>F			CM001263
34	N/A		CGA-TGA	R103>Term			CM1313313
35	N/A		TGC-CGC	C106>R			CM138997
36	N/A		TGC-GGC	C106>G			CM156032
37	N/A		TGC-TGG	C106>W			CM044912
38	N/A		CGG-TGG	R107>W			CM159172
39	N/A		TGC-AGC	C108>S			CM125146
40	N/A		TGC-CGC	C108>R			CM045072
41	N/A		TGC-TAC	C108>Y			CM052270
42	N/A		TGC-TGG	C108>W			HM040053
43	N/A		TGC-TTC	C108>F			CM1714279
44	N/A		CGT-TGT	R110>C			CM971056
45	N/A		TGC-CGC	C117>R			CM078103
46	N/A		TGC-TAC	C117>Y			CM0910785
47	N/A		TGC-TCC	C117>S			CM132414
48	N/A		TGC-TTC	C117>F			CM001264
49	N/A		TCC-TGC	S118>C			HM040099
50	N/A		TGC-TAC	C123>Y	EGF-like 3	CADASIL	CM003013
51	N/A		TGC-TTC	C123>F			CM001265
52	N/A		TGT-GGT	C128>G			CM064156
53	N/A		TGT-TAT	C128>Y			CM023651
54	N/A		TGT-TTT	C128>F			CM125156
55	N/A		GGT-TGT	G131>C			HM070153
56	rs137852642	13740C>T	CGC-TGC	R133>C			CM971057
57	rs137852642	13740C>A	CGC-AGC	R133>S			
58	N/A		TGC-GGC	C134>G			CM156958
59	N/A		TGC-TAC	C134>Y			CM125159
60	N/A		TGC-TGG	C134>W			CM014589
61	N/A		GAT-GGT	D139>V			CM1615017
62	N/A		CGC-TGC	R141>C			CM971058
63	N/A		TTC-TGC	F142>C			CM023652
64	N/A		TGC-TCC	C144>S			CM159357
65	N/A		TGC-TAC	C144>Y			CM001267
66	N/A		TGC-TTC	C144>F			CM003947
67	N/A		TCC-TGC	S145>C			CM044908
68	N/A		TGC-CGC	C146>R			CM971059
69	N/A		TGC-TAC	C146>Y			CM045307
70	N/A		TGC-TGG	C146>W			CM1213543
71	N/A		GGC-GTC	G149>V			CM147538
72	N/A		GGC-TGC	G149>C			CM052271
73	N/A		TAC-TGC	Y150>C			CM001268
74	rs371491165	13794C>G	CAG-GAG	Q151>E			CM0910786
75	rs797045014	13800C>T	CGC-TGC	R153>C			CM971060
76	rs797045014	13800C>A	CGC-AGC	R153>S			
77	N/A		TGC-AGC	C155>S			CM0910788
78	N/A		TGC-TAC	C155>Y			CM125157
79	N/A		TGC-TCC	C155>S			CM044910
80	N/A		TGC-TGG	C155>W			CM159358
81	N/A		TGC-AGC	C162>S	EGF-like 4	CADASIL	CM003014
82	N/A		TGC-CGC	C162>R			CM086704
83	N/A		TGC-TAC	C162>Y			CM170225
84	N/A		TGC-TGG	C162>W			CM035650
85	N/A		GGT-TGT	G165>C			CM0910787
86	rs28933696	13848C>T	CGC-TGC	R169>C			CM961043
87	rs147373451	13852A>G	CAT-CGT	H170>R		CADASIL, Alzheimer’s disease	CM107598
88	rs147373451	13852A>T	CAT-CTT	H170>L		CADASIL, Alzheimer’s disease	
89	N/A		GGT-TGT	G171>C			CM971061
90	N/A		TGC-AGC	C174>S			CM125160
91	N/A		TGC-CGC	C174>R			CM033795
92	N/A		TGC-TAC	C174>Y			CM001269
93	N/A		TGC-TTC	C174>F			CM014211
94	N/A		TCC-TGC	S180>C			CM003015
95	N/A		TTC-TGC	F181>C			CM095734
96	rs28933697	13887C>T	CGC-TGC	R182>C		CADASIL, Alzheimer’s disease	CM961044
97	N/A		TGC-AGC	C183>S			CM001271
98	N/A		TGC-CGC	C183>R			CM001270
99	N/A		TGC-TAC	C183>Y			CM1615080
100	N/A		TGC-TTC	C183>F			CM052272
101	N/A		TGT-AGT	C185>S			CM1010137
102	N/A		TGT-CGT	C185>R			CM971062
103	N/A		TGT-GGT	C185>G			CM014590
104	N/A		TGT-TAT	C185>Y			CM147761
105	N/A		TAC-TGC	Y189>C			CM042442
106	N/A		TGT-AGT	C194>S			CM042443
107	N/A		TGT-CGT	C194>R			CM023653
108	N/A		TGT-GGT	C194>G			CM150660
109	N/A		TGT-TCT	C194>S			CM1010138
110	N/A		TGT-TTT	C194>F			CM001272
111	N/A		TGT-TAT	C194>Y			CM003016
112	N/A		GCG-ACG	A198>T		White matter lesions?	CM0911492
113	N/A		TGT-CGT	C201>R			CM065340
114	N/A		TGT-TAT	C201>Y			CM044914
115	N/A		GCG-GTG	A201>V		CADASIL	CM121679
116	N/A		TGC-CGC	C206>R	EGF-like 5	CADASIL	CM055455
117	N/A		TGC-TAC	C206>Y			CM003017
118	N/A		CGT-CAT	R207>H		White matter lesions?	CM0911493
119	N/A		CGT-TGT	R207>C		CADASIL	CM003018
120	N/A		TGC-AGC	C212>S			CM971063
121	N/A		TGC-CGC	C212>R			CM1714280
122	N/A		TGC-TAC	C212>Y			CM110280
123	N/A		TGC-TGG	C212>W			CM132412
124	N/A		TGC-TTC	C212>F			CM1515586
125	N/A		AGG-AAG	R213>K			CM033796
126	N/A		TAC-TGC	Y220>C			HM0657
127	N/A		TGT-AGT	C222>S			BM1486714
128	N/A		TGT-CGT	C222>R			BM1496778
129	N/A		TGT-GGT	C222>G			CM971064
130	N/A		TGT-TAT	C222>Y			CM023654
131	N/A		TGT-TCT	C222>S			CM106870
132	N/A		TGT-CGT	C224>R			CM159178
133	N/A		TGT-TAT	C224>Y			CM971065
134	N/A		TTT-TGT	F228>C			CM1414770
135	N/A		TGT-AGT	C233>S			CM023655
136	N/A		TGT-CGT	C233>R			CM1010139
137	N/A		TGT-TAT	C233>Y			CM052273
138	N/A		TGT-TGG	C233>W			CM035651
139	N/A		GTG-ATG	V237>M	EGF-like 6	CADASIL	CM025913
140	N/A		TGT-TCT	C240>S			CM052274
141	N/A		TGT-AGT	C245>S			CM056015
142	N/A		TGT-CGT	C245>R			CM052275
143	N/A		TGT-TAT	C245>Y			CM1213544
144	N/A		TGC-AGC	C251>S			CM035643
145	N/A		TGC-CGC	C251>R			CM023656
146	N/A		TGC-GGC	C251>G			CM077212
147	N/A		TGC-TAC	C251>Y			CM092087
148	N/A		GTG-ATG	V252>M			CM150661
149	N/A		TAT-TGT	Y258>C			CM971066
150	N/A		TGC-CGC	C260>R			CM1414771
151	N/A		TGC-GGC	C260>G	EGF-like 7	CADASIL	CM095351
152	N/A		TGC-TAC	C260>Y			CM052276
153	N/A		TGC-TTC	C260>F			CM1213545
154	N/A		TGC-TTC	C271>F			HM060011
155	N/A		TGT-CGT	C278>R		CADASIL	CM155988
156	N/A		TGC-TAC	C291>Y		CADASIL	CM156956
157	N/A		GGT-TGT	G296>C		CADASIL	CM108668
158	N/A		AGC-TGC	S299>C		CADASIL	CM046101
159	N/A		AGA-AAG	G309>K		White matter lesions?	CM0911494
160	N/A		TGC-AGC	C311>S		CADASIL	CM1414772
161	N/A		TGT-TTT	C318>F	EGF-like 8	CADASIL	CM1615081
162	N/A		GTG-ATG	V322>M			CM150662
163	N/A		TGC-AGC	C323>S			CM159171
164	N/A		TGC-TGG	C323>W			CM167812
165	N/A		TGC-TAC	C329>Y			CM1213546
166	rs137852641	14516C>T	CGC-TGC	R332>C			CM014070
167	N/A		TCT-TGT	S335>C			CM052277
168	N/A		TAC-TGC	Y337>C			CM052278
169	N/A		TGT-CGT	C338>R			CM056018
170	N/A		TGT-TCT	C338>S			CM1718752
171	N/A		TGT-TTT	C338>F			CM150663
172	N/A		TGC-TAC	C340>Y			CM1413344
173	N/A		TGC-TTC	C340>F			CM1615082
174	N/A		TGC-CGC	C360>R	EGF-like 9	CADASIL	CM140149
175	N/A		TGT-CGT	C366>R			CM139199
176	N/A		TGT-TGG	C366>W			CM082988
177	N/A		TGT-CGT	C379>R			HM090054
178	N/A		TGT-TCT	C379>S			CM052279
179	N/A		GGC-TGC	G382>C			CM035644
180	N/A		TGT-TAT	C388>Y			CM064155
181	N/A		TGC-CGC	C395>R	EGF-like 10	CADASIL	CM052280
182	rs863225297	16704C>G	TCT-TGT	S396>C			CM125147
183	N/A		TGC-TAC	C408>Y			CM159179
184	N/A		TCC-TGC	S414>C			CM140150
185	N/A		TGC-TGG	C419>W			CM159180
186	N/A		GGT-TGT	G420>C			CM023657
187	N/A		CGT-TGT	R421>C			CM052281
188	N/A		CGC-TGC	R427>C			CM108354
190	N/A		TGT-CGT	C428>R			CM056019
191	N/A		TGT-TAT	C428>Y			CM052282
192	rs267606915	16897T>A	TGT-TCT	C428>S			CM014591
193	N/A		TGT-CGT	C435>R	EGF-like 11	CADASIL	CM035646
194	N/A		TGC-AGC	C440>S			CM056017
195	N/A		TGC-CGC	C440>R			CM052283
196	N/A		TGC-GGC	C440>G			CM023658
197	N/A		TGC-GGC	C446>G			CM1414773
198	N/A		TGC-TCC	C446>S			CM044911
199	N/A		TGC-TTC	C446>F			CM035649
200	N/A		CGC-TGC	R449>C			CM023659
201	rs28933698	16978T>A	TGT-AGT	C455>S			
202	rs28933698	16978T>C	TGT-CGT	C455>R			CM021648
203	N/A		TGT-TAT	C455>Y			CM140151
204	N/A		TGT-TCT	C457>S			CM1010140
205	N/A		TAT-TGT	Y465>C			CM035647
206	N/A		TGC-TAC	C466>Y			CM1111832
207	N/A		TGC-TAT	C478>Y	EGF-like 12	CADASIL	CM150326
208	N/A		TGC-GGC	C484>G		CADASIL	CM125158
209	N/A		TGC-TAC	C484>Y		CADASIL	CM044909
210	N/A		TGC-TTC	C484>F		CADASIL	CM052284
211	N/A		TGC-TAC	C495>Y		CADASIL	CM052285
212	rs11670799	17742C>T	CCC-CTC	P496>L		Ischemic stroke, Alzheimer’s disease, cerebral small-vessel disease	
213	rs114207045	17745C>G	TCG-TGG	S497>W		CADASIL, white matter lesions ?	
214	rs114207045	17745C>T	TCG-TTG	S497>L		CADASIL, white matter lesions ?	CM119547
215	N/A		TGT-CGT	C504>R		CADASIL	CM0911339
216	N/A		TGC-CGC	C511>R		CADASIL	CM052286
217	N/A		TGC-TAC	C511>Y		CADASIL	HM050011
218	N/A		TGC-TTC	C511>F		CADASIL	CM115174
219	N/A		GGC-TGC	G528>C		CADASIL	CM056020
220	N/A		TGC-GGC	C531>G		Leukoencephalopathy, vascular	CM175386
221	N/A		TGC-TCC	C531>S		CADASIL	HM0684
222	N/A		CGC-TGC	R532>C	EGF-like 13	CADASIL	HM070085
223	N/A		TGT-CGT	C542>R			CM140152
224	N/A		TGT-TAT	C542>Y			CM961045
225	N/A		CGC-TGC	R544>C			CM994179
226	N/A		TGC-CGC	C549>R			CM035645
227	N/A		TGC-TAC	C549>Y			CM052287
228	N/A		CGC-TGC	R558>C			CM961046
229	N/A		TGT-TAT	C568>Y			HM0710
230	N/A		TAC-TGC	Y574>C	EGF-like 14	CADASIL	HM0685
231	N/A		ACA-GCA	T577>A			HM0718
232	N/A		CGC-TGC	R578>C			CM961047
233	N/A		TGC-CGC	C579>R			CM121680
234	N/A		CGC-TGC	R587>C	EGF-like 15	CADASIL	CM061879
235	N/A		TGC-CGC	C591>R			CM125164
236	N/A		CGC-AGC	R592>S		White matter lesions?	CM0911495
237	N/A		CGC-TGC	R592>C		CADASIL	CM107182
238	N/A		TGC-TAC	C597>Y			CM1615083
239	N/I		TGC-TCC	C597>S			CM119361
240	N/A		TGC-TGG	C597>W			CM1615018
241	N/A		TGC-CGC	C606>R			CM125148
242	N/A		CGC-CAC	R607>H			CM1615019
243	N/A		CGC-TGC	R607>C			CM003019
244	N/A		TGC-TGG	C617>W			CM1610629
245	N/A		GTC-GTT	V633>V	EGF-like 16	CADASIL	CM124698
246	N/A		CGC-TGC	R640>C		CADASIL	CM125168
247	N/A		GTC-GAC	V644>D		White matter lesions?	CM0911496
248	N/A		GGC-TGC	G667>C	EGF-like 17	CADASIL	CM125169
249	rs10406745	20390G>C	CGC-CCC	R680>P		CADASIL	
250	rs10406745	20390G>A	CGC-CAC	R680>H		CADASIL	
251	N/A		CGC-TGC	R680>C		CADASIL with intracerebral haemorrhage?	CM122007
252	N/A		CCT-ACT	P685>T		CADASIL	CM111340
253	N/A		TAT-TGT	Y710>C	EGF-like 18	CADASIL	CM1313312
254	N/A		CGC-TGC	R717>C			CM1414856
255	N/A		CGC-TGC	R728>C			CM971067
256	N/A		TGC-GGC	C729>G			CM1714281
257	N/A		GTC-GCC	V764>A	EGF-like 19	White matter lesions?	CM119548
258	N/A		TGC-TCC	C775>S	EGF-like 20	CADASIL	CM052288
259	N/A		CGC-TGC	R785>C		CADASIL	CM1413345
260	N/A		GAG-GAA	E813>E	EGF-like 21	CADASIL	CM1615020
261	N/A		TAC-TGC	Y916>C	EGF-like 23	CADASIL	CM1414857
262	N/A		TGT-CGT	C939>R	EGF-like 24	CADASIL	CM125149
263	N/A		GGC-TGC	G953>C	EGF-like 24	CADASIL	CM023660
264	N/A		TGC-TGA	C966>TERM	EGF-like 25	Arteriopathy and cavitating leukoencephalopathy	CM152731
265	N/A		GGC-TGC	G975>C		CADASIL with intracerebral haemorrhage	CM067439
266	N/A		TGC-AGC	C977>S		CADASIL	HM050017
267	N/A		TGC-GGC	C977>G			CM1615021
268	N/A		AGC-CGC	S978>R			HM0711
269	N/A		GCC-TCC	A979>S			CM1313735
270	N/A		TTC-TGC	F984>C			CM003020
271	N/A		CGC-TGC	R985>C			CM971068
272	N/A		TGC-CGC	C986>R			CM1414774
273	N/A		TGC-TAC	C988>Y			CM062927
274	N/A		TGC-TTC	C988>F			CM1714282
275	N/A		TGC-GGC	C997>G			HM070152
276	N/A		TGC-GGC	C1004>G	EGF-like 26	CADASIL CADASIL	CM1714283
277	N/A		TGC-TAC	C1004>Y	EGF-like 26		CM082987
278	N/A		CGC-TGC	R1006>C			CM971069
279	N/A		CCT-TCT	P1008>S			CM148526
280	N/A		GGT-TGT	G1013>C			CM125150
281	N/A		TGC-AGC	C1015>S			CM156030
282	N/A		TGC-CGC	C1015>R			CM994180
283	rs35769976	25217G>T	GCC-TCC	A1020>S			
284	rs35769976	25217G>A	GCC-ACC	A1020>T			
285	rs35769976	25217G>C	GCC-CCC	A1020>P			CM085589
286	N/A		TAT-TGT	Y1021>C			CM023661
287	N/A		TGC-TTC	C1022>F			CM118356
288	N/A		TGG-TCG	W1028>C			CM078549
289	N/A		CGC-TGC	R1031>C			CM971070
290	N/A		GGT-TGT	G1058>C	EGF-like 27	CADASIL	CM014592
291	N/A		TGT-TAT	C1061>Y			CM127977
292	N/A		TAC-TGC	Y1069>C			CM092088
293	N/A		CGT-TGT	R1076>C			CM056016
294	N/A		ACC-TCC	T1098>S	EGF-like 28	CADASIL	CM106871
295	N/A		TGC-TAC	C1099>Y			HM0709
296	N/A		TAC-TGC	Y1106>C			CM1615022
297	N/A		ATG-GTG	M1107>V		Autism spectrum disorder?	CM187124
298	N/A		TGC-TGG	C1131>W	EGF-like 29	CADASIL	CM081358
299	rs112197217	26557C>G	CAC-CAG	H1133>Q		CADASIL	
300	rs112197217	26557C>A	CAC-CAA	H1133>Q		Alzheimer’s disease, ischemic stroke, CADASIL	
301	rs112197217	26557C>T	CAC-CAT	H1133>H		CADASIL	
302	N/A		GGG-CGG	G1134>R		AUTISM -	CM124589
303	N/A		CGC-TGC	R1143>C		CADASIL	CM1715079
304	N/A		CGG-TGG	R1175>W	EGF-like 30	CADASIL -	CM159359
305	rs10408676	26786G>A	GTG-ATG	V1183>M		Alzheimer disease, modifier of?	CM186058
306	N/A		GAC-GAG	D1184>E		CADASIL	CM159173
307	N/A		CGC-TGC	R1210>C			CM1615084
308	rs199638166	26903T>G	TGC-GGC	C1222>G	EGF-like 31	CADASIL	CM1414775
309	rs201680145	26930C>T	CGT-TGT	R1231>C		CADASIL	CM971071
310	rs201680145	26930C>A	CGT-AGT	R1231>S		Alzheimer’s disease	
311	N/A		CAT-CTT	H1235>L		White matter lesions?	CM0911497
312	N/A		TGC-TGG	C1250>W	EGF-like 32	CADASIL	HM090055
313	N/A		TGC-CGC	C1261>R		CADASIL	CM961048
314	N/A		TGC-TAC	C1261>Y		CADASIL	CM052289
315	N/A		CGT-CTT	R1262>L		White matter lesions?	CM119549
316	N/A		TGC-TGG	C1293>W	EGF-like 33	CADASIL	CM186164
317	N/A		TGC-TTC	C1298>F		CADASIL with haemorrhagic strokes	CM135094
318	N/A		TGC-TAC	C1315>Y		CADASIL	CM116899
319	N/A		GCC-ACC	A1450>T	LNR2	White matter lesions ?	CM119550
320	N/A		CTC-CCC	L1515>P	NOD	CADASIL	CM081357
321	rs141320511	31730C>A	CTG-ATG	L1518>M	NOD	White matter lesions?	CM119551
322	rs141320511	31730C>T	CTG-TTG	L1518>L	NOD	White matter lesions, CADASIL, vascular dementia	
323	rs367543285	31734T>C	CTG-CCG	L1519>P	NOD	Migraine and white matter lesions	
324	N/A		CCA-CCG	P1521>P	NOD	Alzheimer disease, modifier of?	CM186056
325	rs78501403	31857G>C	CGG-CCG	R1560>P	NOD	Ischemic stroke, cerebral small-vessel disease	
326	rs78501403	31857G>A	CGG-CAG	R1560>Q	NOD	Ischemic stroke, cerebral small-vessel disease	
327	N/A		GGT-GAT	G1710>D	RAM	White matter lesions?	CM119552
328	N/A		GTG-ATG	V1762>M	RAM	CADASIL	CM1212986
329	N/A		GAC-AAC	D1823>N	NLS	White matter lesions?	CM119553
330	rs115582213	43458G>A	GTG-ATG	V1952>M	ANK	Alzheimer disease (modifier of?)	CM186057
331	rs142007575	44385G>A	GTA-ATA	V2011>1	NLS	Ischemic stroke	
332	rs114447350	44575C>T	CCG-CTG	P2074>L	unknown	Alzheimer’s disease	
333	rs114447350	44575C>A	CCG-CAG	P2074>Q	unknown	Alzheimer’s disease	
334	rs1044009	45022C>G	GCG-GGG	A2223>G	PEST	Alzheimer’s disease, CADASIL	
335	rs1044009	45022C>T	GCG-GTG	A2223>V	PEST	Alzheimer’s disease, CADASIL	

**Table 7:** Mutations in Notch4 associated with neurodegenerative diseases.

Notch4				
A/A	Mutation	Domain	Phenotype	Accession Number
1	EGF-like 6	Schizophrenia	CM099076	EGF-like 6
2	EGF-like 8	Multiple sclerosis	CM133099	EGF-like 8
3	EGF-like 21	Migraine severity	CM134239	EGF-like 21
4	NOD	Migraine duration	CM134237	NOD

**Table 8:** Conserved amino acid changes in cysteine residues at positions (C6, C21, C27, C41, C43, and C52) of the EGF-like repeats of the Notch3 protein, accompanied with the frequency of their appearances and nucleotide changes (marked in red).

A/A		Amino Acid	Frequency	Codons
1	Position 6	C	Given	TGT, TGC
2		R	8	CGT, CGC
3		Y	6	TAT, TAC
4		F	3	TTT, TTC
5		S	3	AGT TCT, AGC, TCC
6		W	3	TGG
7		G	0	GGT, GGC
1	Position 21	C	Given	TGT, TGC
2		R	6	CGT, CGC
3		Y	6	TAT, TAC
4		F	4	TTT, TTC
5		S	2	AGT TCT, AGC, TCC
6		W	2	TGG
7		G	2	GGT, GGC
1	Position 27	C	Given	TGT, TGC
2		R	7	CGT, CGC
3		Y	13	TAT, TAC
4		F	5	TTT, TTC
5		S	7	AGT TCT, AGC, TCC
6		W	4	TGG
7		G	8	GGT, GGC
1	Position 41	C	Given	TGT, TGC
2		R	8	CGT, CGC
3		Y	7	TAT, TAC
4		F	5	TTT, TTC
5		S	6	AGT TCT, AGC, TCC
6		W	1	TGG
7		G	4	GGT, GGC
1	Position 43	C	Given	TGT, TGC
2		R	5	CGT, CGC
3		Y	9	TAT, TAC
4		F	3	TTT, TTC
5		S	5	AGT TCT, AGC, TCC
6		W	3	TGG
7		G	1	GGT, GGC
1	Position 52	C	Given	TGT, TGC
2		R	8	CGT, CGC
3		Y	9	TAT, TAC
4		F	3	TTT, TTC
5		S	6	AGT TCT, AGC, TCC
6		W	4	TGG
7		G	3	GGT, GGC

## Data Availability

No new data were created or analyzed in this study. Data sharing is not applicable to this article.
